# Recent Advancements on the Use of Exosomes as Drug Carriers for the Treatment of Glioblastoma

**DOI:** 10.3390/life13040964

**Published:** 2023-04-07

**Authors:** Angela Galardi, Alexander De Bethlen, Virginia Di Paolo, Silvia Lampis, Angela Mastronuzzi, Angela Di Giannatale

**Affiliations:** Department of Pediatric Hemato-Oncology and Cell and Gene Therapy, IRCCS, Bambino Gesù Children’s Hospital, 00165 Rome, Italy

**Keywords:** glioblastoma, exosomes, therapy

## Abstract

Glioblastoma (GBM) is the most common and aggressive cancer of the brain. Presently, GBM patients have a poor prognosis, and therapy primarily aims to extend the life expectancy of affected patients. The current treatment of GBM in adult cases and high-grade gliomas in the pediatric population involves a multimodal approach that includes surgical resection followed by simultaneous chemo/radiotherapy. Exosomes are nanoparticles that transport proteins and nucleic acids and play a crucial role in mediating intercellular communication. Recent evidence suggests that these microvesicles may be used as biological carriers and offer significant advantages in targeted therapy. Due to their inherent cell-targeting properties, circulation stability, and biocompatibility, exosomes are emerging as promising new carriers for drugs and biotherapeutics. Furthermore, these nanovesicles are a repository of potential diagnostic and prognostic markers. In this review, we focus on the therapeutic potentials of exosomes in nano-delivery and describe the latest evidence of their use as a therapeutic tool in GBM.

## 1. Introduction

Glioblastoma (GBM) is a highly aggressive tumor that arises from astrocyte cells. It is characterized by a fast growth rate, high malignancy, the ability to infiltrate surrounding normal brain tissue, and a high degree of genetic and microenvironmental heterogeneity [1]. GBM is the most common primary brain tumor in adults, accounting for 45.6% of primary malignant brain tumors [2], and typically presents at a median age of 64 years [3] but can occur at any age, including childhood; however, pediatric high-grade gliomas are rare, only representing approximately 5% of all pediatric brain tumors [4]. GBMs are classified as either primary (de novo) or secondary, depending on their origin: GBMs developed with no known precursor are primary, whereas GBMs evolved from low-grade tumors are secondary [5]. In most cases, GBM is detected as a primary tumor, and increasing age is a negative indicator of prognosis. Patients characteristically display early symptoms, including headaches and intracranial pressure, as well as progressive focal neural deficits such as problems with movement, sensation, vision, or speech as a result of necrosis of the brain tissue [6]. Progression of the disease is often accompanied by seizures and worsening of other symptoms [6]. At present, GBM cannot be cured, and therapy mainly aims to extend the life expectancy of affected patients; as such, there is an important need to evaluate the current mode of GBM treatment and evaluate new promising opportunities to better address this disease [7]. Research is being carried out to understand the pathways that lead to the development of GBM in order to develop more personalized approaches to therapy [8]. An emerging field in GBM research is represented by the study of extracellular vesicles (EVs), which are nanostructures produced by normal and cancerous cells and play a role in intercellular communication [9]. Studies have shown that GBM cells release a large number of exosomes, which help promote the hallmarks of cancer. Since exosomes are specific to their parent cells, isolating them to evaluate their contents can give insight into the development of GBM [10]. Moreover, exosomes prove to be useful markers for diagnosis and prognosis. In this review, we will overview the relevance of exosomes in GBM and discuss the most recent studies, suggesting the importance of exosomes as potential drug-delivery vehicles for novel therapeutic approaches.

### 1.1. Pediatric-Type Diffuse High-Grade Gliomas

High-grade gliomas are rare in children, although cases do occur and are characterized by distinct genetic mutations. Four pediatric subtypes of pediatric-type diffuse high-grade gliomas, distinguishable from their genetic profiles, have been newly identified in the fifth edition of the WHO Classification of Tumors of the Central Nervous System (WHO CNS5) [11]

✓ Diffuse midline glioma H3 K27-altered, diffuse hemispheric glioma H3 G34-mutant;
✓ Diffuse pediatric-type high-grade glioma H3-wildtype;
✓ IDH-wildtype;
✓ Infant-type hemispheric glioma.

In pediatric high-grade gliomas, which occur largely de novo, isocitrate dehydrogenase 1 (*IDH1*), epidermal growth factor receptor (*EGFR*), phosphatase and tensin homolog (*PTEN*), and other classic driver mutations and alterations identified in adult GBM are rare [12]. This suggests that the nature of pediatric high-grade gliomas may differ compared to the more common later onset of the disease. In a gene-expression profiling study carried out by Haque and colleagues [13], it was found that overexpression of Y-box protein 1 (*YB-1*) was common in most pediatric high-grade gliomas, unlike adult cases. Additionally, studies analyzing gene expression and copy number variation in pediatric gliomas have shown significant biological differences between pediatric high-grade gliomas and adult GBMs, notably the infrequent presence of amplified *EGFR* or its recurrent gain-of-function variant (*EGFRvIII*) in pediatric cases compared to adult [12]. On the other hand, platelet-derived growth factor receptor A (*PDGFRA*) amplification appears to be a common feature of pediatric high-grade gliomas, possibly suggesting an early initiating role of this mutation in pediatric tumorigenesis [14]. Immunotherapeutic approaches are in development for pediatric high-grade gliomas, involving the use of therapeutic vaccination to redirect T-cells against tumor antigens [15]. However, the effectiveness of immunotherapeutic treatment is still to be thoroughly evaluated.

### 1.2. GBM Current Therapy and Limitations

Currently, the treatment of GBM in adult cases and high-grade gliomas in the pediatric population involves a multimodal approach that includes surgical resection followed by chemotherapy and simultaneous radiotherapy. Maximal surgical resection has been shown to benefit patients by lessening tumor burden and relieving mass effect [16]. Consequently, the goal of surgery is currently to strive for complete resection of the detectable tumor. Various surgical procedures are now available to obtain more complete resection with fewer post-operative complications; for example, gamma knife radiosurgery can precisely eliminate cell tissue without the need for surgical incision [17]. Work is also being done to optimize resection through the use of intraoperative visualization techniques such as high-resolution ultrasound to confirm the completeness of resection and high-resolution preoperative magnetic resonance imaging data for neuronavigation [16].

Nevertheless, there are still limitations to the efficacy of surgery in treating patients. Although surgical resection is correlated with improved patient outcomes, complete tumor resection is almost impossible to achieve due to the invasive nature of GBM and its rapid spread to crucial regions of the brain [16]. Consequently, recurrence of the tumor is common even post-surgery [18,19], and various other complications, such as the risk of infection and post-operative functional deficits, remain important limitations to overcome if surgery is expected to garner better results in the future.

Following surgery, the use of temozolomide (TMZ) as first-line chemotherapy has been the standard practice ever since its FDA approval in 2005 [20]. TMZ is an oral alkylating agent whose mechanism of action is based on its ability to bind to the DNA of cancer cells, inducing a DNA-damage response that can ultimately lead to impaired genetic material and cell death. Specifically, TMZ is metabolized in the body to its active form, 5-(3-methyltriazen1-yl) imidazole-4-carboxamide (MTIC). MTIC further reacts with water to produce 5-aminoimidazole-4-carboxamide (AIC) and the highly reactive methyldiazonium cation. This cation can methylate the O6 guanine residues on DNA, forming a toxic lesion (O6-MeG). TMZ toxicity is, therefore, primarily mediated via O6-MeG, as this methyl mark can lead to DNA strand breaks, replication fork collapse, and ultimately cell death [21].

However, the effectiveness of TMZ in treating GBM is restricted by several factors. The major limitation is that over 50% of GBM patients treated with TMZ develop resistance [22] through several mechanisms, such as the activation of repair enzymes such as O6-methylguanine-DNA methyltransferase (MGMT). MGMT can repair the DNA damage caused by TMZ by removing the methyl groups added to DNA [23]. As such, in GBM, high levels of MGMT expression are associated with poor response to TMZ treatment. MGMT activity can be regulated at the epigenetic level through hyper/hypomethylation of the MGMT gene promoter region. Hypomethylation is associated with increased enzyme activity and correlates positively with TMZ resistance and negatively with patient outcomes [22]. Consequently, the epigenetic status of MGMT has been established as a surrogate marker of intrinsic resistance to TMZ [23].

Combination therapy using TMZ with MGMT-targeting drugs has been proposed for TMZ-resistant patients; however, MGMT inhibitors can cause further complications as the enzyme is also required for normal DNA repair of healthy cells [24]. Additionally, glioma cells can activate survival pathways, such as the Wnt/β-catenin and PI3K/Akt/mTOR signaling pathways, which can protect the cells from drug-induced apoptosis [25]. This can lead to treatment failure and poor patient outcomes.

Regrettably, even following surgery and concomitant radio/chemotherapy, the median survival time for patients is only approximately 16 months [26]. Evidently, the current approach is hindered by several limitations, which render long-term success unlikely.

In addition to the limitations of surgery and chemotherapy described previously, the development of new GBM drugs and treatments is also challenging for having to overcome obstructions such as the genetic variability of the disease (which makes the development of gene therapies difficult) and the physiological location of the tumor, as novel drugs must be able to cross the blood–brain barrier (BBB). As such, the advent of new delivery vehicles, such as exosomes and liposomes, that allow specific targeting of drugs at the tumor site may evolve to be a staple in GBM treatment as they have the potential to allow increased delivery and even co-delivery of multiple drugs targeting the tumor site directly. In this review, we reported the most recent findings on the role of exosomes as nano-carriers and their use as a therapeutic tool in GBM (Summarized in Table 1).

## 2. Exosomes: Biogenesis, Release, and Functions

Exosomes are nanovesicles, approximately 30–100 nm in diameter. They are characterized by a spheroidal bilayer membrane, consisting of a phospholipid bilayer of endocytic origin (sphingomyelin, cholesterol, and ceramides) able to influence their transport, structure, release, and their signaling and membrane proteins (which also confer class specificity), such as members of the tetraspanin family (CD9, CD63, CD81), the endosomal sorting complex required for transport (ESCRT), Alix and flotelin, actin, integrins, major histocompatibility complex I and II (MHCI; MHCII) [39]. They are secreted by various cell types, including cancer cells, and are present in biofluids such as blood, cerebrospinal fluid (CSF), and urine [40]. Exosomes carrying proteins, lipids, DNA, and RNA are secreted into the extracellular space and internalized by target cells [41]. Exosome formation occurs when the membrane of the multivesicular endosomes (MVEs) swells inward and pinches off to generate small membranous vesicles within the MVEs [42]. When MVEs fuse with the plasma membrane, exosomes are released into the extracellular environment [43]. Due to the mechanism of biogenesis, the exosomal membrane has the same orientation as the parental cell plasma membrane, and it is enriched in endosome-related membrane transport and fusion proteins, lipids, and tetraspanins [44]. Since exosomes are released by almost all cells, both under physiological and pathological conditions, their molecular content can vary significantly [45]. Once released into the extracellular space, transmembrane ligands on the surface of the exosome can interface directly with the surface receptors of the recipient cell, triggering a downstream signaling cascade to activate it [46]. At this point, the exosome can fuse with the cell membrane, and its content is released inside the cell [47]. Exosomes have mostly been characterized in immune cells (dendritic cells, B cells, T cells, and macrophages) and tumors [48]. Internalization of exosomes by tumor cells affects cellular pathways and several cancer hallmarks, including reprogramming of stromal cells, modulating immune responses, reconstructing extracellular matrix architecture, or even endowing tumor cells with drug-features resistance [49]. Recent studies have shown that GBM cells produce numerous exosomes, which carry materials that enable the cells to promote tumor invasion and migration, reduce the body’s immunity against the tumor, chemoresistance, and tumor neoangiogenesis. The exosomes, being unique to the parental cells, can provide a more comprehensive understanding of GBM biogenesis when isolated [50]. Research has also confirmed that exosomes found in the plasma of GBM patients contain the mRNA of the mutated/variant *EGFRvIII*, which is a distinctive feature of a clinical subtype of GBM [51]. Al-Nedawi et al. showed that GBM cells could transfer the *EGFRvIII* receptor to other cells through exosomal DNA, inducing pro-angiogenic qualities, suggesting that they play a role in initiating angiogenesis [52].

## 3. Strategies for the Use of Exosomes as Nano-Carriers

**Figure 1:**
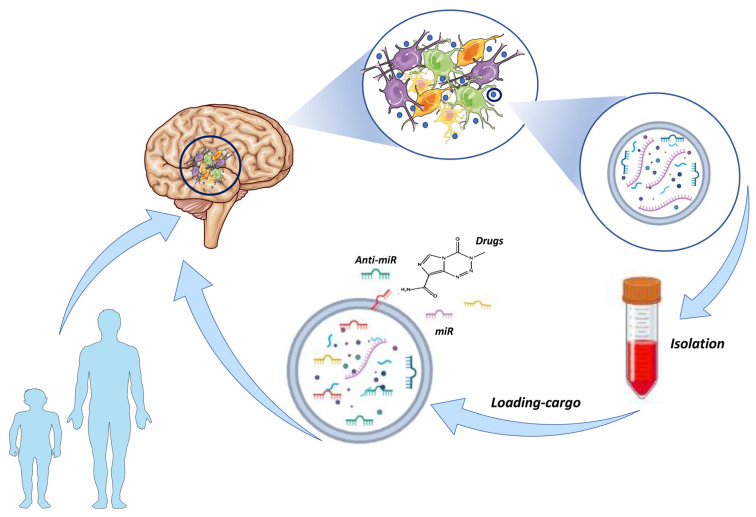
Exosome-based therapy workflow: Outline of the steps typically involved in developing novel therapies based on exosomes derived directly from GBM tumors. GBM cells secrete exosomes that carry a variety of proteins and nucleic acids. These exosomes can be isolated and modified by loading cargoes such as anti-tumor drugs and anti-microRNA (miRNA)s. In this way, the advantages of exosomes, such as their ability to cross the BBB and target specific cells based on their surface proteins, can be used to direct therapy to the tumor site. Consequently, exosome vehicles may help to overcome chemoresistance in GBM.

The advantages of exosomes as nano-carriers are their stability and ability to pass through the BBB [53], making them effective for drug delivery to the brain. Additionally, these organic microvesicles are less toxic than alternative synthetic counterparts [54]. Consequently, exosomes show promise for encapsulating chemotherapeutic drugs and can play a role in targeted therapy, reducing chemoresistance and systemic side effects [55] (Figure 1).

### 3.1. Current Method of Exosome Isolation

**Figure 2:**
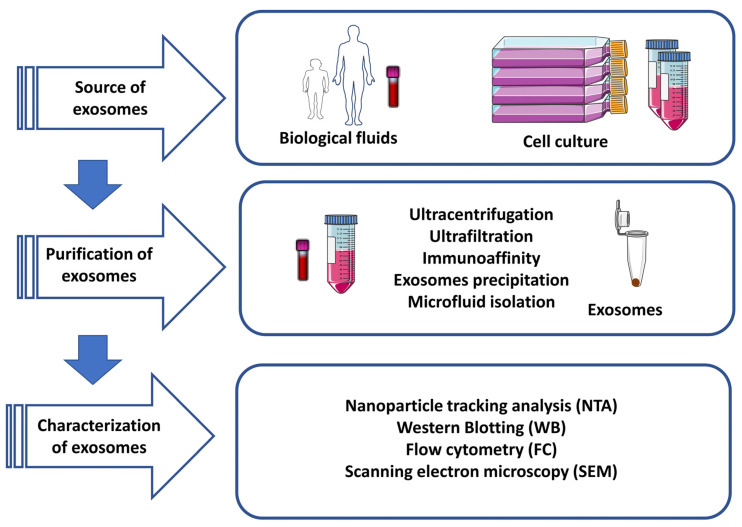
Techniques for exosome isolation, purification, and characterization.

Exosome isolation remains challenging due to exosomes’ small size and low density [56]. Ideal isolation methods should be quick, efficient, reliable, and affordable, using easily collected samples. To date, five isolation techniques have been developed [57]; these include ultracentrifugation, ultrafiltration, immunoaffinity, precipitation, and microfluidics. There are advantages and disadvantages for each technique; however, the gold standard for exosome isolation is ultracentrifugation, which first uses low-speed centrifugation to eliminate cells and debris, and then high-speed centrifugation to pellet the exosomes [58]. However, this process is time-consuming, and exosomes could be damaged during the high-speed centrifugation process [59]. Ultrafiltration is a size-dependent method that is faster and cheaper than ultracentrifugation but with the drawback of low specificity, as particles of similar size to exosomes are also filtered [60]. Alternatively, immunoaffinity isolation has high specificity thanks to the identification of specific exosomal membrane-bound proteins that can be targeted with specially created antibodies operating as ligands but is very expensive [61,62]. This method provides highly purified exosomes and the possibility of subtyping. However, it is very expensive, and the number of isolated exosomes is low. Exosomes precipitation techniques are simple but require incubation overnight as well as pre- and post-clean-up to remove non-exosomal contaminants. These techniques use water-excluding polymers—namely polyethylene glycol (PEG)—to alter exosomes’ physicochemical properties, such as solubility and dispersibility, to force exosomes to settle out [63]. Finally, the latest techniques, including microfluidics technologies, allow for rapid and efficient microscale exosome isolation based on physical and biochemical properties (e.g., size, density, and immunoaffinity), but the major drawback is a lack of standardization and validation for this technique [64] (Figure 2).

### 3.2. Strategies for Exosome Loading

Exosomes can be loaded with physical materials and used as a drug-delivery system to treat diseases and tumors. The exosome surface can be modified with homing molecules, such as ligands, pH-responsive motifs, and magnetic materials, to provide targeting properties for in vivo delivery. There are multiple techniques used to load therapeutic or diagnostic cargo, including incubation, transfection, physical treatment, and in situ assembly and synthesis. Incubation is a passive loading method where exosome-producing cells are incubated with the desired cargo. If the cargo is hydrophobic, it can diffuse across the cell and exosomal membranes spontaneously [65]. This method can also be classified as active if the drug is loaded after exosome isolation. Factors that influence passive loading include the solubility, size, and charge of the drug molecule [66]. Transfection is an active loading method where desired nucleic acids are loaded into exosomes using a vector [67]. This technique provides high drug-loading efficiency but is limited by the toxicity of the transfection agents [68]. Physical treatment methods include electroporation and sonication. Electroporation uses an electric field to alter cell membranes and transfer DNA or drugs into exosomes [65,66,67,68,69,70]. However, this method may damage the structural integrity of the vesicles and result in adverse events [71]. Sonication uses sound waves to alter the cell membrane permeability of exosomes but may result in significant changes in the structure and integrity of the exosome population. Freeze–thaw treatment, extrusion, dialysis, and surfactant treatment are further physical treatments that also enhance cargo loading into exosomes [72,73,74]. Hypotonic dialysis has been shown to increase drug-loading efficiency into exosomes by more than 11 times [72]. Finally, in situ assembly and synthesis techniques promote metal nanoparticle loading by reducing metal ions into nanoparticles within exosomes [44]. The main advantage of this strategy is the maintenance of exosome integrity, but its applications are limited due to technological barriers.

### 3.3. Exosome Modification for Targeted Delivery

Normally, exosomes spread through extracellular spaces and biofluids by diffusion and can be internalized by recipient cells. To deliver exosome content to specific tissues or cells, the destination must be targeted specifically. One way to achieve this is through surface engineering, which modifies the exosomes’ surface profile to act as a hallmark for the receiving cell-type uptake [75]. Surface engineering can be achieved through genetic engineering or chemical modification. Genetic engineering involves the coupling of ligands or homing peptides to transmembrane proteins produced on exosome surfaces. The resulting modified exosomes have targeting ligands on their surfaces and are produced by donor cells transfected with plasmids encoding fusion proteins [68]. On the other hand, chemical modification uses chemical substances to bind to the exosomes through various techniques, such as click chemistry [45], formation of triazole linkage resulting in azide formation, linkage with PEG and Distearoyl phosphoethanol amine analogs that allow exosomes to bypass their elimination by the immune system with prolonged plasma time for activity [76]. However, the last technique causes instability of the exosomes and their aggregation during manipulation. The chemical modification enables the display of a wide range of natural and synthesized ligands via conjugation processes or lipid assembly. Another way to target exosomes is by utilizing the specific chemical properties of different tissues or cells. This can include a pH gradient-driven targeting strategy or the surface charge or lipophilicity of the exosomes. Owing to the high rate of intracellular glycolysis and lactate production, the tumor microenvironment is acidic compared with normal tissues, which makes pH-responsive drug-delivery systems a promising platform to target tumors and reduce the side effects of drugs [77]. This pH gradient-driven targeting strategy can be combined with a ligand-receptor binding strategy to enhance specificity and delivery efficiency. The surface charge or lipophilicity of exosomes is another critical property that affects cellular internalization, in vivo distribution, and the targeting efficiency toward desired tissues/cells [78]. Although surface charge does not confer similar specificity to exosomes as ligand-receptor binding, it can be a useful supplement to increase the targeting efficiency of exosomes to certain organs.

Additionally, targeted delivery can be achieved with the assistance of external attractive forces, such as magnetism [79]. For example, the targeting ability of exosomes can be further promoted by conferring magnetic attraction and ligand-receptor-binding capacity to engineered exosomes. Jia et al. packaged superparamagnetic iron oxide nanoparticles (SPIONs) into exosomes and conjugated the exosomal membrane with neuropilin-1-targeted peptide (RGERPPR, RGE) by click chemistry [64]. These doubly modified exosomes cross the BBB smoothly, target glioma specifically, and transfer chemotherapeutic drugs efficiently, therefore enhancing targeted imaging and therapeutic effects of glioma. These pilot studies explored the possibility of magnetic-dependent targeted delivery of exosomes, laying the theoretical and experimental foundation for its future application in disease diagnosis and treatment.

### 3.4. Application in Brain Tumor

The vast majority of ongoing exosome-based clinical trials aim to identify diagnostic or prognostic biomarkers; however, due to their characteristic properties in delivering functional cargo to diseased cells, a rapidly increasing number of trials are also investigating exosomes as therapeutic agents in a wide range of diseases, including cancer.

Several small molecules, both hydrophobic and hydrophilic, have been incorporated into exosomes. In most cases, exosomal delivery leads to higher drug accumulation in target cells, improved stability, and blood circulation time, thus improving the potency of small-molecule drugs and lowering the half-maximal inhibitory concentration (IC50) [80]. As such, exosomes may help overcome the main hurdle in the treatment of brain tumors; low efficiency of drug delivery to glial cells due to the BBB [81].

In a study by Zhuang et al., the effects of curcumin-loaded exosomes on glial cells were investigated using a non-invasive (intra-nasal) route of administration to treat inflammation related to brain cells [82]. This strategy may be considered a novel therapeutic approach in the treatment of neurological disorders. In an alternative study, the transport of anti-tumor drugs Doxorubicin (DOX) and Paclitaxel (PTX) by exosomes across the BBB was investigated. In this work, the fluorescent dye Rhodamine was used to tag the drugs and observe their selective uptake into the brain when loaded into exosomes [31]. Jia et al. expanded on this work by engineering exosomes through conjugation with RGERPPR, and RGE, after loading with DOX and SPIONs, to target brain cells in glioma [83].

In these experiments, increased uptake efficiency of engineered exosomes with higher therapeutic function due to a synergistic effect was observed, providing a new approach for improving the diagnosis and treatment of brain tumors.

## 4. Use of Exosomes in the Treatment of GBM

### 4.1. Regulation of miRNA

**Figure 3:**
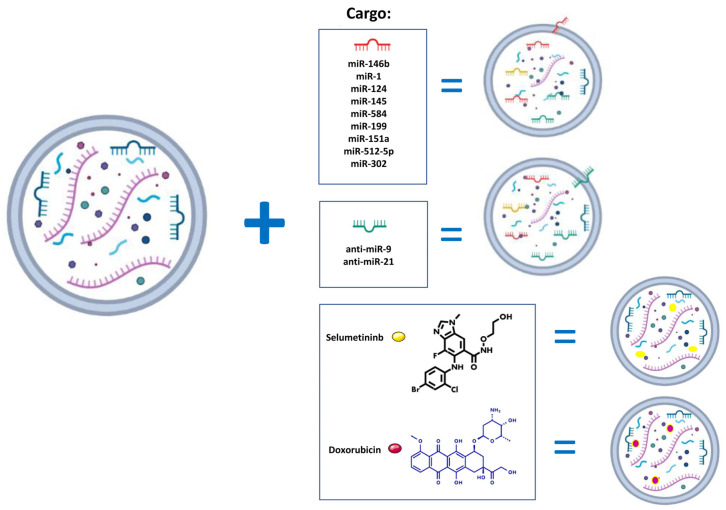
Schematic illustration of the process of engineering exosomes with the cargoes described in the text (miR, anti-miR, and drugs).

Research has shown that failure to regulate miRNAs can increase the likelihood of GBM development by promoting angiogenesis and the invasive and proliferative behavior of glioma cells [84]. Moreover, miRNAs can influence several processes in GBM cells, such as chemoresistance and functional drug efflux. Through the targeting of specific genes, miRNAs can behave functionally as either tumor suppressors or oncogenes [85]. To date, exosomes derived from mesenchymal stem cells (MSCs) transfected with anti-tumor miRNAs have shown potential as a promising therapeutic approach for the treatment of gliomas and GBM [86] (Figure 3).

### 4.2. Transport of miRNA Mimics

If miRNAs can function as oncosuppressors, providing exogenous miRNA mimics could be an effective strategy in fighting cancer. However, there is a limited amount of research showing the effects of the re-introduction of miRNAs into target cells within the tumor environment in GBM [30]. Early research dating back to 2013 describes MSCs’ ability to release large amounts of exosomes and integrate endogenous miRNA mimics into GBM cells, providing an efficient pathway for therapeutic miRNA delivery [27,28]. For example, Katakowski and colleagues tested MSC exosomes as a vehicle for miRNA release in malignant glioma. The miR-146b overexpressed in MSC exosomes (M146-exo) was tested both in vitro and in vivo [27]. In vitro results showed that the presence of exosomes derived from MSCs expressing miR-146b reduced proliferation and invasion of 9L glioma tumor cells. In vivo results demonstrated that injection of M146-exo into Fischer rats carrying 9L gliosarcoma significantly reduced tumor volume and improved animal survival [27]. In an investigation with a similar approach, Lee and colleagues focused on the delivery of miR-124 and miR-145, as these miRNAs are expressed in low levels in GBM cells. Synthetic mimics of miR-124 and miR-145 were efficiently delivered to GBM cells by exosomes and were able to modulate the gene expression of recipient cells, for example, by decreasing the migration of glioma cells in vitro and in vivo [28]. Subsequently, it was confirmed that treatment with Exo-miR124a can inhibit the growth and clonogenicity of patient-derived GBM stem cells and can treat mice harboring intracranial GBM stem cell xenografts. Mechanistic studies have shown that miR-124a acts by reducing the expression of Forkhead Box Protein A2 (*FOXA2)*, a known target of miR-124a and that apoptotic cell death correlates with *FOXA2*-mediated abnormal accumulation of intracellular lipids [87]. Tumor-suppressive functions of miR-1 have been shown in numerous human malignancies [88,89]. In GBM, miR-1 deficiency has been described as an important factor in tumor growth, neovascularization, and invasiveness by simultaneously targeting major components of oncogenic signaling networks: *JN K*, *PRCs*, *MET*, *EGFR*, *Annexin A2 (ANXA2*). The re-introduction of miR-1 into GBM EVs can reverse tumor malignancy and remodel the tumor environment [30]. GBM development could also be prevented by introducing miR-584 to GMB cells. This miRNA acts as a tumor suppressor by binding the 3′UTR of Pituitary Tumor-Transforming Gene 1 Protein-Interacting Protein (*PTTG1IP)*, blocking its expression and activity [90]. This was demonstrated by Kim et al., who used exosomes derived from MSCs transfected with miR-584 to affect the proliferation and migration of U-87 cells in vitro and decrease tumor mass weights in U-87 xenograft nude mouse model (the treatment did not affect the body weight of the animals) [33]. Chemoresistance is an obstacle in the fight against various types of cancer, including GBM. Recently, exosomes have been proposed in addressing this problem, as they can help restore drug sensitivity [91]. For example, attenuation of malignant gliomas by down-regulation of ArfGAP with GTPase domain, ankyrin repeat, and PH domain 2 (*AGAP2*), which is a target of miR-199, has been reported. Because overexpression of miR-199a inhibits migration, invasion, and proliferation of U251 cells, miRNA mimic 199a has been packaged into the exosomes of MSCs, which are taken up by GBM cells, exerting an anti-tumor effect as well as increased chemosensitivity to TMZ [36]. Likewise, to explore the role of miR-151 and its target, X-Ray Repair Cross Complementing 4 (*XRCC4)* in TMZ-resistant GBM, cells were transfected with miR-151a to effectively load exosomes with therapeutic miR-151a. The data demonstrated conclusively that exogenous miR-151a loading into exosomes prevented them from conferring TMZ resistance to recipient cells and suggested therapeutic potential when used in conjunction with TMZ chemotherapy. Additionally, miR-151a expression in CSF-derived exosomes may serve as a substitute for diagnostic markers during biopsy profiling to assess the effectiveness of treatment in GBM patients [34]. Yan et al. [37] showed that exosomes produced by bone MSCs (BMSCs) modify tumor characteristics by releasing miR-512-5p. MiR-512-5p was downregulated in GBM tissues and cells, and Jagged 1 (JAG1) was the target gene of miR-512-5p. miR-512-5p could be contained and transported by BMSC-derived exosomes to GBM cells in vitro. By reducing *JAG1* expression, BMSC-derived exosomal miR-512-5p reduced GBM cell growth and brought about cell cycle arrest. These in vitro results were confirmed in vivo, with BMSC-exosomal miR-512-5p extending mouse longevity and reducing GBM growth. These findings imply that miR-512-5p is delivered into GBM by BMSC-derived exosomes, which then target *JAG1* to inhibit the tumor’s growth [37]. It has been found that the miRNA cluster miR-302-367 has tumor-suppressive properties and may be valuable in GBM treatment [32]. Fareh et al., sought to develop a cell-based therapy by exploiting the ability of glioma cells to secrete exosomes containing RNA molecules [92]. GBM cells were engineered to stably express miR-302-367. Following this modification, it was observed that neighboring GBM cells displayed altered proliferation, tumorigenicity, and expression of stemness markers. Furthermore, implantation of cells expressing miR-302-367 exosomes in combination with GBM stem cells altered tumor development in the brains of mice, indicating therapeutic potential [32].

### 4.3. Transport of Anti-miRNA

Although it seems clear that miRNAs are often involved in the inhibition of tumor development processes and thus are “oncosuppressors” sometimes miRNAs can also behave as “oncogenes”. An important example is offered by miR21, which plays a pivotal role in GBM pathogenesis. miR-21 can affect a variety of cellular and molecular pathways such as insulin-like growth factor (IGF)-binding protein-3 (*IGFBP3*), Reversion Inducing Cysteine Rich Protein With Kazal Motifs (*RECK)*, and TIMP Metallopeptidase Inhibitor 3 (*TIMP3)*, with the final result of promoting GBM tumorigenesis [93]. Moreover, exosomal miR21 levels in the CSF have been proposed as a promising indicator for GBM diagnosis and prognosis, particularly with values to predict tumor recurrence or metastasis [94,95]. These findings provide a basis for blocking the effect of miR-21 in the tumor microenvironment as a possible therapeutic approach to combat progression and invasion. Monfared and colleagues [35] constructed a miR-21 sponge and packaged it into secretory exosomes. When the engineered exosomes were introduced to u87-MG and C6 cells, down-regulation of miR-21 and upregulation of its targets, Programmed cell death 4 (*PDCD4*) [96], and *RECK* (which are key regulators of apoptotic and metastatic pathways) were observed. To confirm their results, they also used the C6 cell line to generate rat xenograft models that demonstrated the efficacy of the manipulated exosomes in suppressing tumor growth and inducing tumor growth retardation. The latter result was even more evident when freshly prepared exosomes were used in vivo experiments were conducted using both freshly prepared engineered exosomes and using stored exosomes. However, although both treatments showed a good effect in inhibiting tumor growth, fresh exosomes are more effective in vivo applications resulting in the eradication of the tumor mass. Munoz et al. instead focused on miR-9 molecules that have been shown to suppress the mesenchymal differentiation of GBM cells. They identified an increase in miR-9 concentration in TMZ-resistant GBM cells, which is involved in the expression of the drug efflux transporter P-glycoprotein. On this basis, they showed that reversed chemoresistance of GBM cells to TMZ occurred by targeting anti-miRNA through MSCs. To block miR-9, they tested an anti-miR-9-Exosome obtained from transfection with human bone marrow-derived MSC. When U87 and T98G cells were treated with anti-miR-9-Cy5 (15 and 30 nM) in combination with TMZ (200 uMol/L) showed an increase of caspases activities and a significant decrease in viability (*p* < 0.05) compared with TMZ treatment alone [29] (Figure 3).

### 4.4. Exosomes as Drug Delivery System

The value of exosome engineering by drug loading for the treatment of GBM, as well as other cancers, has been investigated by several authors. The molecular properties of exosomes generated from human GBM-astrocytoma U-87 MG and brain endothelial bEND.3 cell lines were emphasized by Yang et al. for their capacity to interact and cross biological barriers. According to their findings, bEND.3-derived exosomes enabled greater internalization of a fluorescent marker in bEND.3 cells via an energy-dependent internalization mechanism. Furthermore, CD63 tetraspanin transmembrane proteins, which are overexpressed in brain endothelial cells, were thought to be the cause of this active process, which is receptor-mediated endocytosis. To transfer PTX or DOX through the BBB in a zebrafish model of brain tumor by using U-87 MG glioma, they reported the utilization of both U-87 MG and bEND.3 exosomes. Freely administered DOX and PTX are unable to cross the BBB, but the vesicle-packaged tool made it possible, slowing the growth of tumors [31]. In a study by Zhang et al., which also investigated DOX delivery, the potential of exosome-coated DOX-loaded nanoparticles (ENPDOX) in BBB penetration, inducing immunogenic cell death (ICD) and promoting survival of GBM-bearing mice, was investigated. DOX-loaded nanoparticles (NPDOX) were coated with exosomes prepared from mouse brain endothelial bEnd.3 cells. ENPDOX was taken up by bEnd.3 cells and could penetrate the BBB both in vitro and in vivo. Furthermore, in vitro ENPDOX induced apoptosis and ICD of glioma GL261 cells. Systemic administration of ENPDOX resulted in the maturation of dendritic cells, activation of cytotoxic cells, altered production of cytokines, suppression of proliferation, increased apoptosis of GBM cells in vivo, and prolonged survival of GBM-bearing mice. These findings suggest that ENPDOX may be a potent therapeutic strategy for GBM which warrants further investigation in clinical application [97]. Selumetinib is a drug used to treat young patients with recurrent or refractory low-grade glioma [98]. Since NF type 1, selumetinibs’ primary target, is often significantly mutated in GBM, Lee and colleagues used it as a prospective treatment for GBM [38]. They showed that the ability of selumetinib-loaded U87MG-derived exosomes (U87-Selu exo) to target their parent cells could be exploited for targeted GBM (U87MG cell) therapy. Even at higher dosages, U87-Selu exo did not exhibit any cytotoxicity to healthy brain cells or damage to the liver or kidney in vivo. Therefore, according to these findings, GBM with a U87MG origin is specifically affected by the anticancer effects of U87-Selu exo. The fact that U87-Selu exo is not harmful to healthy liver and brain cells suggests that they are effective therapeutic options for the treatment of GBM. Furthermore, because the BBB is present in the central nervous system, it is currently difficult to prove if GBM-derived exosomes have an in vivo homing impact. As a result, additional research is necessary to examine the in vivo homing effect of glioma-derived exosomes using a suitable brain tumor model, such as an orthotopic model [38]. The engineering of exosomes by both drug loading and surface functionalization has also been tested recently. Lee et al. have developed methotrexate (MTX)-loaded EVs functionalized with therapeutic [Lys-Leu-Ala (KLA)] and targeted [low-density lipoprotein (LDL)] peptides. Compared to blank EVs, in vitro investigations showed that EVs decorated with LDL or KLA-LDL could improve uptake by human primary GBM cell line U87 and permeation into three-dimensional GBM spheroids. As a result, the payload’s therapeutic effect is improved. EV extravasation over the BBB and distribution in the glioma site were both clearly promoted by peptide LDL, according to imaging experiments conducted both ex vivo and in vivo [99] (Figure 3).

### 4.5. Exosomes as Drugs

To date, only one study that investigates the use of exosomes derived from rat bone marrow MSCs GBM has been published, making it the first time that exosomes are used as a drug, not just a drug carrier [100]. Briefly, rat bone marrow MSCs were extracted, and their effect on the rat GMB cell line was studied. Different exosome concentrations were cultured with C6 cells, and their effects were assessed in vitro at various time intervals. Results demonstrated that isolated exosomes primarily induce apoptosis as a mechanism of cell death, and a linear relationship between exosome content and cytotoxicity was found. Through the scratch test, colony formation test, and transwell experiment, it was later demonstrated that exosomes had a major impact on the behavior of C6 cells during migration and invasion. Analysis of the scratch of C6 cells after being cultured with different concentrations of exosomes (20, 40, and 80 ug/mL) at 0, 6, 12, 24, and 48 h post-seeding showed that exosomes are able to reduce cell mobility. Specifically, after 24 h, all concentrations show a reduction in the closure, while at 48 h, only at the highest concentration is about 20% of the scratch area not filled by C6 cells. Additionally, in the Transwell assay, exosomes reduced C6 migration and invasion behavior at 24 h at 80 ug/mL. So, controlling cell migration and proliferation prevents tumor growth and reduces the possibility of new tumors developing at other sites.

## 5. Conclusions

Considering the low survival rate among patients affected by GBM, with currently approved treatments, new therapeutic approaches are urgently needed. In the last decade, exosomes have emerged as a promising carrier for drug delivery due to their low immunogenicity and strong biocompatibility. Several studies, also in GBM, have demonstrated that exosomes can be used as vehicles for drugs and nucleic acids or be engineered for anti-tumor treatment. However, various limitations are associated with the use of exosomes as carriers of anti-tumor molecules. These limitations include efficient isolation and purification, lack of a suitable standardized loading strategy, and tumor targeting ability that enable to obtain of a high-quality amount of standardized exosomes. The improvement of targeting specificity could be overcome by the improvement of the targeting ability with refined techniques that allow anchoring targeting proteins on the exosome membrane. Moreover, an important challenge in using an exosome-based drug-delivery system is a deep understanding of the biological mechanisms of these vesicles. In this context, further biological studies in this field are needed in order to allow a large-scale use of a nanoplatform for cancer therapy.

## Figures and Tables

**Table 1:** Current GBM exosome-based treatment.

Exosome Cargo	Loading Method	Model of GBM (vitro/vivo)	Outcomes	Refs.
miR-146b	Transfection	in vitro: 9L in vivo: 9L-derived xenografts	↓ tumor growth ↓ tumor volume	[27]
miR-124	Transfection	in vitro: U87, A172 in vivo: U87-derived xenografts	↓ migration ↓ of target gene CDK6 inducing differentiation of GBM stem cells in vivo	[28]
miR-145	Transfection	in vitro: U87, A172 in vivo: U87-derived xenografts	↓ migration and glioma stem cell self-renewal ↓ of GBM stem cell self-renewal	[28]
anti-miR9	Transfection	in vitro: U87, T98G	TMZ chemosensitivity restored	[29]
miR-1	Transfection	in vitro: U87, U251, U373, Gli36 in vivo: U87-derived xenografts	↓cell-to-cell and cell-to-matrix adhesion, reduced neurosphere sizes, and inhibited in vitro invasion ↓ tumor growth, neovascularization, and invasiveness	[30]
Doxorubicin	Incubation	in vitro: U87 in vivo: zebrafish xenotransplantation	↑ intracellular uptake and cytotoxicity of anticancer ↓ tumor volume compared to drug alone	[31]
miR-302	Transfection	in vitro: GSC primary cultures (TG1, TG6, and GB1)	↓ the number of mitotic cells and impaired their ability to be clonal	[32]
		in vivo: TG1-derived xenografts	↓ Cyclin D1, Cyclin A, E2F1 expression, and the CXCR4 pathway compromising stemness properties and inhibiting tumor development in vivo	
miR-584	Transfection	in vitro: U87	↓ expression of CYP2J2 and significant decrease in cell proliferation	[33]
		in vivo: U87-derived xenografts	↓ tumor mass without reduction in body weight	
mir-151a	Transfection	in vitro: R GBM1	Recovered TMZ sensitivity in TMZ-resistant cells	[34]
		in vivo: U251-derived xenografts	Restored TMZ response of resistant cells	
miR-21-sponge	Transfection	in vitro: U87, C6	↓ of miR-21	[35]
miR-199	Transfection	in vitro: U251	↓ proliferation, migration and invasiveness; ↑ TMZ chemosensitivity	[36]
		in vivo: C6-derived xenografts	Retardation and suppression of tumor growth	
miR-512-5p	Transfection	in vitro: U87	Induced cell cycle arrest	[37]
Selumetinib	Electroporation	in vitro: U87 in vivo: U87-derived xenografts	↓ cancer cell proliferation ↓ tumor volume	[38]

## Data Availability

Not applicable.
